# Poverty determinants of acute respiratory infections among Mapuche indigenous peoples in Chile's Ninth Region of Araucania, using GIS and spatial statistics to identify health disparities

**DOI:** 10.1186/1476-072X-6-26

**Published:** 2007-07-02

**Authors:** Flavio Rojas

**Affiliations:** 1 Department of Biostatistics, School of Public Health, University of North Carolina at Chapel Hill, USA

## Abstract

**Background:**

This research concerns *Araucanía*, often called the Ninth Region, the poorest region of Chile where inequalities are most extreme. *Araucanía *hasn't enjoyed the economic success Chile achieved when the country returned to democracy in 1990. The Ninth Region also has the largest ethnic *Mapuche *population, located in rural areas and attached to small agricultural properties. Written and oral histories of diseases have been the most frequently used methods to explore the links between an ancestral population's perception of health conditions and their deprived environments. With census data and hospital records, it is now possible to incorporate statistical data about the links between poverty and disease among ethnic communities and compare results with non-*Mapuche *population.

**Data sources:**

Hospital discharge records from Health Services North N = 24,126 patients, year 2003, and 7 hospitals), Health Services South (N = 81,780 patients and 25 hospitals); CAS-2/Family records (N = 527,539 individuals, 439 neighborhoods, 32 *Comunas*).

**Methods:**

Given the over-dispersion of data and the clustered nature of observations, we used the global Moran's I and General G Gettis-Ord procedures to test spatial dependence. These tests confirmed the clusters of disease and the need to use spatial regression within a General Linear Mixed Model perspective.

**Results:**

Health outcomes indicate significantly higher morbidity rates for the *Mapuche *compared to non-*Mapuche *in both age groups < 5 and 15–44, respectively; for the groups 70–79 and 80 + years of age, this trend is reversed. Mortality rates, however, are higher among *Mapuches *than non-*Mapuches *for the entire Ninth Region and for all age groups. Mortality caused by respiratory infections is higher among *Mapuches *than non-*Mapuches *in all age-groups. A major finding is the link between poverty and respiratory infections.

**Conclusion:**

Poverty is significantly associated with respiratory infections in the population of Chile's Ninth Region. High deprivation areas are associated with poverty, and poverty is a predictor of respiratory infections. *Mapuches *are at higher risk of deaths caused by respiratory infections in all age groups. Exponential and spherical spatial correlation models were tested to estimate the previous association and were compared with non-spatial Poisson, concluding that significant spatial variability was present in the data.

## Background and overview

This article addresses some aspects of the relationship between poverty and disease. The study area is located in Chile's Ninth Region, also known as *Araucanía*, the poorest of the country's 13 regions, and one where income distribution reveals inequality that is not only the worst in the country, but the worst in the world, with a Gini coefficient of 0.58 [1,2]. The main goal of this article is to show the link between the region's neighborhoods to the health of the residents of the region and to illustrate the role poverty and deprivation play in communicable infectious diseases of the respiratory system. In addition, this article will discuss how this relationship is established within certain areas in the region. Finally, given the ethnic history of *Araucanía *and the high percentage of *Mapuches *in the population, this research article will test whether this ancestral population, which bears the highest poverty rates, is more vulnerable to disease compared to non-*Mapuches*. These ethnic differences may provide important clues to understanding the differential mortality rates between *Mapuche *and non-*Mapuche *populations.

For more than a decade, Chile has been praised internationally for its sound economic and social policies and the stability of the macroeconomic strategies put in place after the dissolution of military rule. Stable economic growth with social investments during the last 16 years are seen by many as key factors for the country's success in reducing poverty, which at the end of the military dictatorship had impacted close to 5.5 million people. Despite these important achievements, income distribution has remained unchanged, and certain regions of Chile are still profoundly primitive. In a country that has experienced increasing economic prosperity, the *Araucanía *region continues to display two challenging conditions: the highest rates of poverty and high inequality in income distribution. Another essential aspect of the *Araucanía *region is its ethnicity: More than one-third of the population is of *Mapuche *ancestry. In some rural areas, the percentage of *Mapuche *population is even higher.

Two issues arise from the inequality of income and the backward living conditions in the *Araucania *region. On a theoretical level lies the question of the transition of a market economy into a fully integrated, global, world-trade economy and the new economy's ability to trickle-down its wealth to the primitive areas of the country. A second issue of this transition is of a social nature: the extent to which reducing poverty on national, aggregate level still leaves some areas lagging behind, specifically how the existence of areas with high levels of poverty and high levels of inequalities, particularly in income and education, affect health and well-being in the population. Last but not least, there are oral histories and *Mapuche *descriptions of respiratory infections, timing, and prevalence that do not necessarily correspond with Western-medicine predictions. "Intercultural epidemiology as a discipline to study occurrences of disease in populations of a different culture as well as how a discipline incorporates own categories and etiologies of disease from a contextual perspective and from a particular culture. As an example, the study of death of *Mapuche *children less than 5 years old as a consequence of respiratory infections in a year is observed as a trend to peak in the months between September-March. From biomedical perspective it is claimed that environmental risk factors would be less important during that time of the year. However, in September it is the beginning of scarcity or lack of food; the crops of previous year are gone and there is nothing to eat, sell or trade and the market does not have yet new crops"[3].

This article is an effort to provide original, fully detailed data to highlight the health conditions in the population of Chile's Ninth Region and test statistically whether there is an association between poverty and poor health. Several studies have challenged the nature and direction of this association [4-6]; other studies have responded with the multilevel nature of the income inequality hypothesis [7,8]. The statistical modeling in this paper uses a different strategy to link poverty and health by combining spatial statistics and Geographical Information Systems (GIS) with individual poverty records and hospital discharge records (patient records) and then aggregating them by neighborhoods. We hope that this methodological strategy may overcome some limitations of using self-rated health as an indicator for peoples' health condition on one hand, and income inequality as a contextual factor for poverty on the other.

## Methodology

### The study area

**Figure 1:**
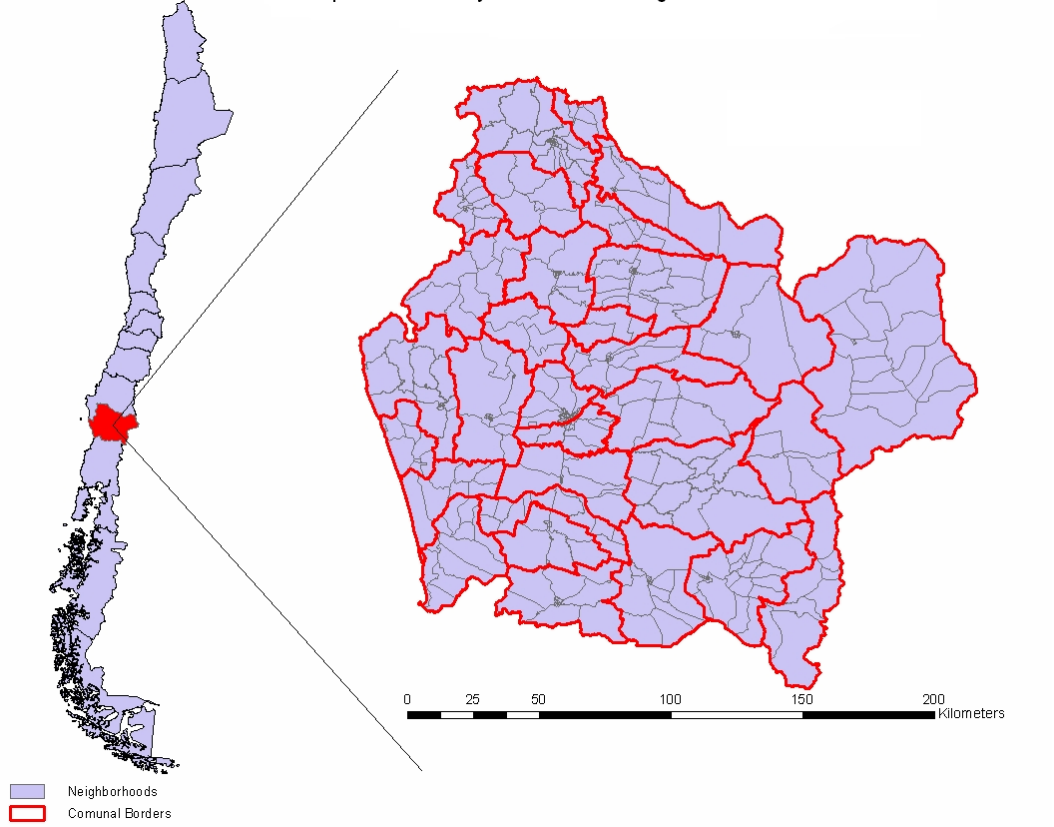
Map of Chile and Study Area: Chile's Ninth Region of Araucanía.

The *Araucanía *region covers 31,814 km^2 ^and, with 869,535 inhabitants, has a density of 27 habitants per square kilometer, according to the 2002 Population Census. This region accounts for 5.8% of Chile's population. A total 203,221 people declared themselves to be *Mapuches*, which is 23.4% overall regionally, but in some *Comunas *this percentage is as much as 64.6% (Puerto Saavedra) or 59.6% (Galvarino). The study area involves all 32 *Comunas *and 439 neighborhoods ranging in size from 65 mt^2 ^(the smallest) to 803.8 km^2 ^(the largest), with 72.5 km^2 ^as average (Figure 1). The household level information was generated from CAS-2 poverty records; a total of 527,539 individuals and 94,131 households with their detailed socio-economic situation were later linked to a GIS-generated base-map. The study area covering the health system involves two health services, *Araucanía *North and *Araucanía *South, with 25 public hospitals under their administration and seven private hospitals under their supervision. The total number of hospital discharge records for the year 2003 was 105,906 patients, 12.2% of the population. Of those, 64,099 had complete identification codes; 49,893 individuals were unique people, and the remaining 14,206 patients had multiple hospital discharge records ranging from 2 to 12 hospitalizations. Finally, the census data includes information at the *Comuna *level, detailing age-groups, ethnicity, and gender for all the population of year 2002.

### The GIS data

A geo-referenced base-map for the neighborhoods and boundaries of each and every *Comuna *was obtained from the regional government. Additional geographic information and the system of coordinates for the *Araucanía *region were obtained from the Military Geographic Institute (IGM). This database contained the geographic names and other features (e.g. rivers, roads, indigenous reservations, cities, towns, villages, volcanoes) for every known place in the region. From a total of 5,552 geographic places, 1,360 were selected for being considered "human settlements," regardless of their size. In addition, centroids for each neighborhood area were generated using the *add XY coordinates *option from ArcMAP/ArcGIS software. Once these geographic coordinates were generated for each neighborhood in the GIS, we proceeded to merge the hospital discharge records containing the unique identification number (*Rol Unico Tributario*, RUT Unique Tax Code Identifier) of every patient, with the corresponding RUT of each applicant in CAS-2/Family records. In the end, by matching the geo-referenced neighborhood poverty records with hospital records, we were able to set up a seamless master data set for the spatial analysis of respiratory communicable infections. A total of 49,190 hospital patients were identified by their households as a result of this spatial merging.

### The attribute data

We used a combination of four variables to represent the poverty score (variable "score2"). This variable is the resulting sum of averaged scores for "education attained," "quality of the dwelling," "family income," and "employment" for the family members who are applying to any subsidy or social support through CAS-2 records. The computed scores allow a ranking of applicants so that social workers can establish priorities: the lower the score, the poorer the family and thus the higher their priority in access to the social network of government assistance. In addition, every member has to provide his or her unique national identification (RUT) and precise address to be considered. The data on diseases was obtained from medical hospital discharge records and categorized according to the burden of disease (BOD) methodology [9]. As a result, 32 categories of diseases were created into which the patient population from the hospital system was seamlessly absorbed. Age groups were organized according to BOD methodology as follows:

(in years) 0–4 ;  5–14;   15–29 ;  30–44;   45–59;   60–69 ;  70–79;   80+ 

Ethnicity was established according to either of the patients' last names as belonging to or not belonging to ancestral culture. This method follows "surname analysis" which uses an individual's last name to estimate the likelihood that the individual belongs to a particular racial or ethnic group [10]. This method is used routinely in the hospital records in Chile and in the U.S. Census Bureau to identify Hispanics. Although there are no current estimations in Chile of the accuracy comparing self-reporting ethnicity (Census) and surname analysis based on either last name, in the United States, the 1990 Census Hispanic list showed an overall sensitivity of 79% and a specificity of 90% compared with self-reported ethnicity in a national sample [10]. Gender attributes as male or female were also included as well as the *Comuna *of residence. Nearly 10% of the patients are treated for respiratory infections (J00-J06, J10-J18, J20-J22 and H65-H66 of ICD-10 codes). Because of the high incidence of respiratory infections in the total number of patients, we decided to select this category and link it with poverty records. We also selected respiratory infections on a theoretical basis, since poverty and respiratory infections were found to be related in other native populations in North America, including Native Americans in Alaska, Alberta, and Saskatchewan [11]. Other considerations for including respiratory infections were based on their incidence as poverty-related deaths among Third World countries. Acute Respiratory Infection (ARI) is the second largest cause of deaths (9.9% of all deaths) among high mortality, low income countries [12,13].

Integrating attribute data was first accomplished through incidence rate standardizations. This concept offers a mechanism to adjust summary rates to remove the effect of known risk factors (such as age, ethnicity, and gender) and make rates from different populations comparable [14]. Poverty records in combination with hospital discharge records were first linked using patients' unique national identification number, the RUT. Once the dataset linking 12 months of patients' hospitalizations with their socio-economic/poverty condition was operational, we proceeded to incorporate totals from Census 2002 data by age-group, ethnicity, gender, and *comuna *of origin, thus obtaining incidence rates by individual and by individuals grouped by neighborhood. Similar steps were taken to link death certificate records, causes of deaths, age, ethnicity, gender and *comuna *with census data in order to generate standardized rates for mortality for the population of *Araucania*.

Table 1 displays the results of direct standardization methods applied to hospital discharge records and mortality rates for the entire health system of the *Araucania *Region in the year 2003. The upper part of the table shows that morbidity rates per thousand are highest in people younger than 5 years and those 80 years and older with values 186.9 [95% CI 183.1 to 190.8] for non-*Mapuches *under age 5 and 227.7 [95% CI 218.5 to 237.1] for *Mapuches *under age 5, and 398 [95% CI 386.8 to 409.5] for non-*Mapuches *and 332.4 [95% CI 307.3 to 359.1] for *Mapuches *in all diseases considered (Table 1, lower part). These values are also statistically significant for specific diseases such as respiratory infections where similar Relative Risks are found in the younger group and the older group of 80 years and older (Table 2). Another important conclusion from the data is that unequal rates of diseases between *Mapuche *and non-*Mapuches *begin early in life for all diseases considered and also for respiratory infections. However, these inequalities are reversed in the 80 + years old group. In both situations, the standardized values are significant.

**Table 1:** All diseases, overall morbidity and mortality rates compared by age groups and ethnicity. Directly standardized rates per 1,000. Ninth Region of Araucanía, Chile 2003 *

			Mapuche	C.I. 95%		Non-Mapuche	C.I. 95%	
			
	Age Groups	Rates	Lower	Upper	Rates	Lower	Upper	RR
Morbidity	< 5	227.7	218.5	237.1	186.9	183.1	190.8	1.24
N = 105,906	5 – 14	55.7	52.8	58.7	58.6	57.2	60.0	0.98
	15 – 29	121.5	117.8	125.3	112.4	110.7	114.1	1.10
	30 – 44	120.7	116.4	125.1	112.6	110.8	114.3	1.09
	45 – 59	109.0	103.8	114.4	112.9	110.7	115.2	0.99
	60 – 69	179.7	168.9	191.0	176.7	172.5	181.0	1.05
	70 – 79	279.9	262.1	298.6	291.5	284.8	298.3	1.00
	80 >	332.4	307.3	359.1	398.0	386.8	409.5	0.88

Mortality	< 5	8.47	6.67	10.61	2.99	2.55	3.48	2.83
N = 5,509	5 – 14	0.46	0.25	0.79	0.26	0.18	0.36	1.79
	15 – 29	1.61	1.18	2.16	0.83	0.69	0.99	1.94
	30 – 44	2.56	1.95	3.30	1.74	1.54	1.97	1.47
	45 – 59	8.65	7.21	10.29	4.94	4.49	5.41	1.75
	60 – 69	21.69	17.91	26.03	14.36	13.23	15.56	1.51
	70 – 79	51.84	44.79	59.68	34.56	32.37	36.86	1.50
	80 >	145.85	127.68	165.89	107.42	101.72	113.36	1.36

*Source: Own, based on Health Services Araucanía North and South Hospital discharge records

**Table 2:** Respiratory infections, morbidity and mortality rates compared by age groups and ethnicity. Directly standardized rates per 1,000. Ninth Region of Araucanía, Chile 2003 *

			Mapuche	C.I. 95%		Non-Mapuche	C.I. 95%	
			
	Age Groups	Rates	Lower	Upper	Rates	Lower	Upper	RR
Morbidity	< 5	91.7	86.2	97.4	75.1	72.7	77.6	1.22
N = 14,394	5 – 14	11.0	9.8	12.3	13.5	12.8	14.2	0.82
	15 – 29	2.6	2.2	3.1	3.4	3.1	3.7	0.77
	30 – 44	3.9	3.2	4.6	3.9	3.6	4.3	0.98
	45 – 59	8.6	7.3	10.1	7.5	6.9	8.1	1.14
	60 – 69	29.2	24.9	34.0	19.3	17.9	20.8	1.51
	70 – 79	57.1	49.3	65.7	49.4	46.6	52.3	1.16
	80 >	96.2	83.9	109.9	100.8	95.1	106.7	0.95

Mortality	< 5	0.39	0.06	1.26	0.18	0.08	0.34	2.20
N = 259	5 – 14	0.00	0.00	0.19	0.00	0.00	0.06	.
	15 – 29	0.02	0.00	0.14	0.01	0.00	0.03	4.17
	30 – 44	0.00	0.00	0.19	0.04	0.01	0.08	0.00
	45 – 59	0.11	0.03	0.32	0.06	0.02	0.13	1.98
	60 – 69	0.61	0.17	1.55	0.36	0.21	0.59	1.67
	70 – 79	2.00	0.75	4.29	1.33	0.93	1.84	1.50
	80 >	22.07	14.51	32.18	8.14	6.62	9.91	2.71

*Source: Own, based on Health Services Araucania North and South Hospital discharge records

Respiratory infections, ICD-10 Codes, J00-J06, J10-J18, J20-J22, H65-H66

The CIs followed the gamma distribution and were calculated following Fay-Feuer method for the upper limits and the Anderson-Rosemberg method recommended by NCHS for lower limits [15,16]. This method is also used in Harvard's Geocoding Project [17].

Although it is not possible to explain why non-indigenous, senior aged groups reverse the previous unequal pattern of disease among *Mapuche *children and adolescent/young aged, (Tables 1 and 2 upper parts) we have found that, in all causes of deaths and in mortality rates caused by respiratory infections (Tables 1 and Table 2 lower parts), the worst outcome of disease-mortality-is much higher for *Mapuche *people in all age groups compared to non-*Mapuche*. In sum, although morbidity is somewhat higher among *Mapuche *children and young adults, with cyclical elements in midlife groups, overall rates of disease resulting in death are significantly higher across all *Mapuche *age-groups when compared with non-*Mapuches*. Tables 1 and 2 lower parts, last column indicates that the relative risk of dying from respiratory infections for those < 5 years old is almost twice the relative risk of experiencing the same disease. Table 1, reports that the relative risk of dying from any disease is more than twice the relative risk of experiencing any disease. If *Mapuche *death rates are higher in all age-groups, and their relative risks exceeds the non-*Mapuches*, it becomes particularly important that future research should also address the issue of birth rates and survival rates among ancestral peoples and their capacity to prevent their own extinction.

Tables 1 and 2 also confirm that the pattern and rates of mortality are quite similar to the pattern of morbidity rates for all diseases and specific diseases considered, with death rates caused by acute respiratory infections being significantly higher for *Mapuches *than non-*Mapuches *in all age groups.

### Data categorization and manipulation

From the above data, it is evident that there are differential rates of diseases and risk that give the *Mapuche *population higher mortality rates than the non-*Mapuche *population. If this is the case, what then is the link between poverty and specific diseases such as respiratory infections? We categorized here the poverty variable following official definitions [18-20]. Since the information was obtained at individual levels, it was possible to collapse the data using spatially referenced neighborhood centroids (points) at the sub-*Comuna *area level. Poverty records also carried the unique identification code for each person applying for social benefits. This unique national ID code was later linked to individual patient hospital discharge records. Merging procedures using SAS software allowed us to generate seamlessly a single, geo-referenced database with a poverty/disease record per individual. Standardized morbidity rates adjusted for sex and ethnicity were later generated; afterwards, individual incidence rates for the ARI were aggregated at neighborhood level. Individual incidence rates were added by neighborhood areas. The most important aspect in this data manipulation and identification of standardized rates by neighborhoods was the possibility of finding spatially clustered patterns of respiratory infections.

**Figure 2:**
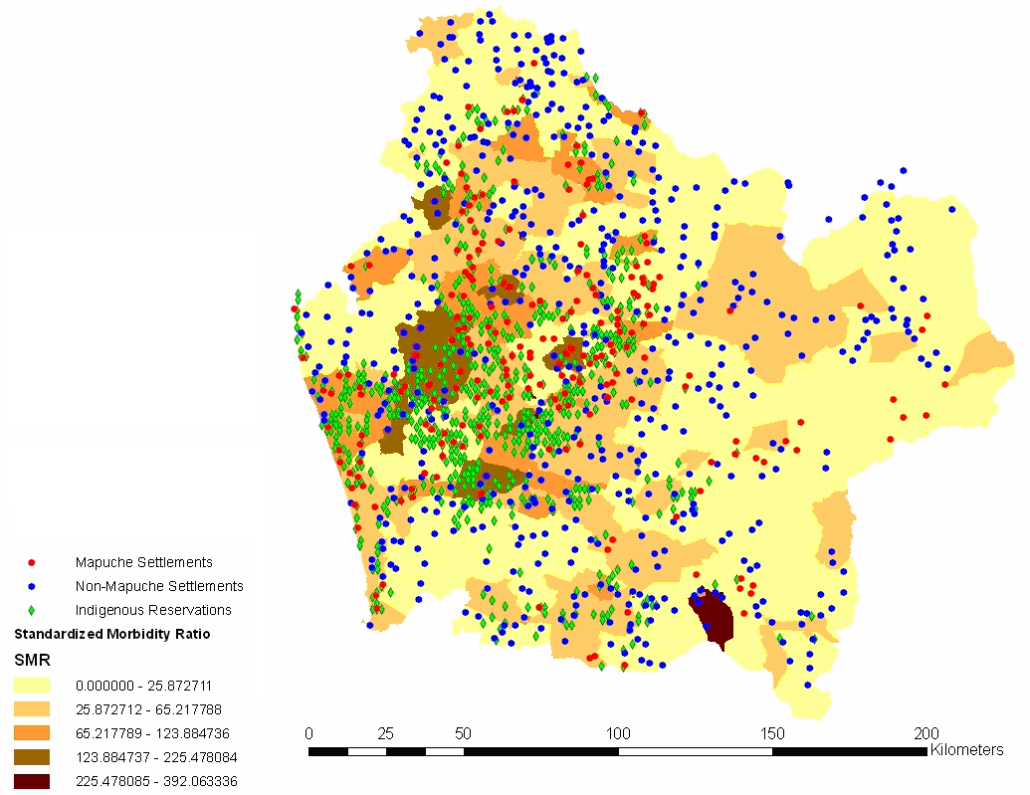
Respiratory Infections SMR 2003 in the 439 neighborhoods of Araucanía Region.

**Figure 3:**
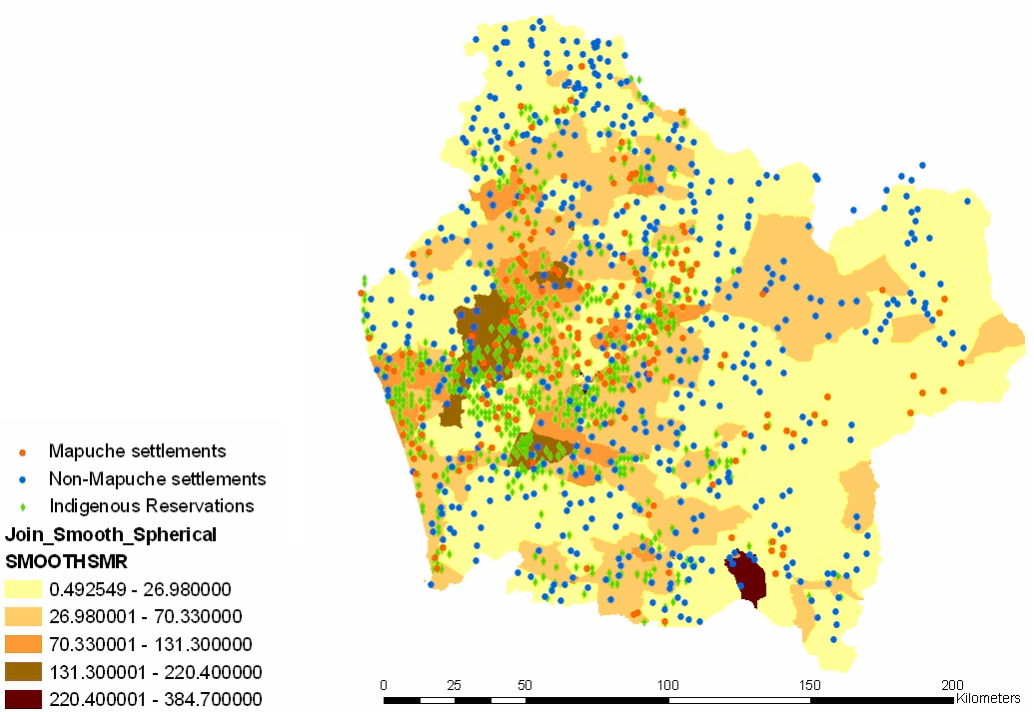
Smoothed respiratory infections SMRs using the spatial GLMMS Araucanía Region.

**Figure 4:**
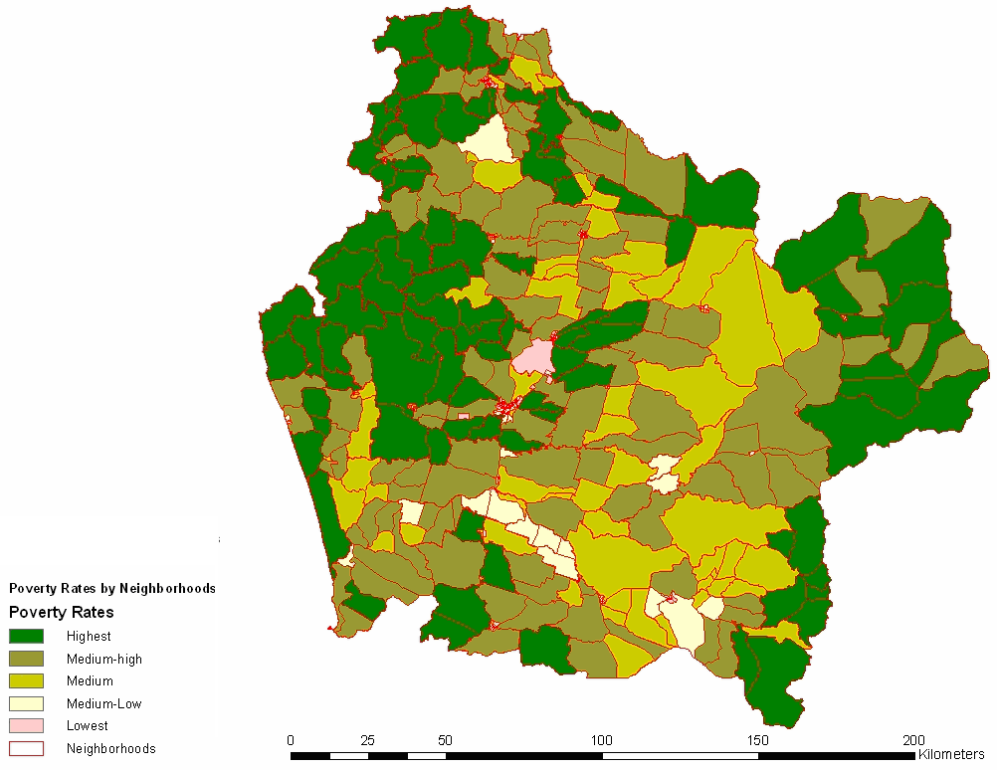
Poverty rates in the 439 neighborhoods of Araucanía Region.

Figure 2 displays original standardized morbidity rates across neighborhoods and populations (*Mapuche *versus non-*Mapuche*) and indigenous reservations identified with data of their locations from the Military Geographic Institute (IGM). Note that the map presents a scattered plot with blue dots representing non-*Mapuche *settlements. While the highest standardized rates are clearly clustered, non-*Mapuche *dwellings are dispersed, with some of these dwellings falling within the highest area rates and other dwellings being located in areas with lower rates. Since the variability in the estimated local rates is based on populations of very different sizes, some rates may be more accurately estimated than others, and this may obscure spatial patterns of disease risk. Rates based on small populations or on small numbers of disease cases are likely to be elevated artificially, reflecting lack of data than a true elevated risk [14]. As a way to assess this potential problem, we have introduced spatial smoothing. Figure 3 incorporates the conditional, smoothed SMR map based on the Generalized Linear Mixed Models (GLMM) (spherical) model. The smoothed SMR map obtained is consistent with Figure 2; the values in the lowest and upper categories however move slightly above 0 and below 392. The larger rates tend to occur in more rural areas toward the coast and the mountains, which is also where the higher poverty rates are predominant. In sum, it may be said that non-*Mapuche *dwellings seem to have no spatial cluster in any of the standardized areas. A different pattern is found, however, with *Mapuche *settlements (red dots) and indigenous reservations (green diamonds). There is some dispersion, but observations are clearly clustered on the highest and medium-high SMR scores. Figure 4 displays poverty areas overlayed to previous population dwellings. Note that highest poverty rates are also clustered, and while non-*Mapuche *dwellings seem to show a scattered pattern, *Mapuche *settlements are concentrated in neighborhoods with the highest levels of poverty according to CAS-2 records.

At this point, it is important to test spatial autocorrelations. This test will verify whether the map visualization and color representation that illustrates a geographically bounded group of occurrences (of sufficient size and concentration) occurred by chance [21]. Using the Moran's I test (to measure the extent to which neighboring areas tend to have similar values); in this case the index will be positive. If neighboring regions tend to have different values, then the index will be negative, but when there is no correlation between adjacent values, the expected value tends toward 0. In addition to Global Moran's I, we introduced Getis-Ord General test to test high-low clustering.

Table 3 shows the results of Moran's and Getis-Ord General tests. The z values for both suggest that the spatial clustering pattern is very strong and that spatial dependence among neighborhoods is also statistically significant. With Moran's I *p-value *of 0.0015, it is very unlikely that adjacent values of respiratory infections are the result of a random pattern. G tests are also highly significant, with a *p-value *of .0001 – meaning that the clustered patterns found have only a very slight chance of being the result of random chance.

**Table 3:** Global Clustering Indexes for Neighborhood-level. Respiratory Infection Incidence Rates

Index Statistics		Respiratory Infections
Moran's I	Value	0.0398
	Expected	-0.0022
	Variance	0.0001
	z-score	3.7976*
General G	Value	3.5052
	Expected	2.6110
	Variance	3.5101
	z-score	4.7727**

* Significant at: 0.0015

** Significant at <.0001

Statistically, the main implication of having positively tested clustered configurations is the existence of deviations from the Poisson distributions [14,21]. Generalized Linear Mixed Models (GLMM) have been used to handle Poisson distributions with overdispersion [21]. Other authors have suggested a more flexible distribution, such as a negative binomial in the case of overdispersed data, or adjusting the covariance matrix of a Poisson-based analysis with a scaling factor [24].

Spatial statistical models are also appropriate tools to use when positive associations are found in neighboring observations. In the next section, we will first introduce several models for handling clustered data and, second, test the relationship between poverty and respiratory infections.

### Statistical Analysis

A long debate has been going on as to whether or not income inequalities and/or poverty affect the population's health [25-27]. It has been argued that the studies that do not support this relationship have either measured inequality in areas too small to report real social differences, or have been conducted in countries that are more egalitarian such as Sweden, Japan, Canada, Denmark, and New Zealand [25]. Contextual and even global factors also may have intervening effects which alter the original and usual controversial relationship between poverty and precarious health [25,26]. Some authors who point out those methods suggest that multilevel approaches, rather than national ones, are more appropriate for disaggregating individual-contextual effects in populations' health [27,28].

As previously stated, this research has observed high numbers of clusters among individual patients, thus violating a statistical analysis based on assumptions of normality, independence, and homogeneity in which ordinary linear regression models are based. Therefore while

*y *= *xβ *+ *ε*

represents the simple linear regression model, by applying a Log function we use a loglinear model [24]. for a rate:

LogμN=β0+β1x
 MathType@MTEF@5@5@+=feaafiart1ev1aaatCvAUfKttLearuWrP9MDH5MBPbIqV92AaeXatLxBI9gBaebbnrfifHhDYfgasaacH8akY=wiFfYdH8Gipec8Eeeu0xXdbba9frFj0=OqFfea0dXdd9vqai=hGuQ8kuc9pgc9s8qqaq=dirpe0xb9q8qiLsFr0=vr0=vr0dc8meaabaqaciaacaGaaeqabaqabeGadaaakeaacqWGmbatcqWGVbWBcqWGNbWzdaWcaaqaaGGaciab=X7aTbqaaiabd6eaobaacqGH9aqpcqWFYoGydaWgaaWcbaGaeGimaadabeaakiabgUcaRiab=j7aInaaBaaaleaacqaIXaqmaeqaaOGaemiEaGhaaa@3C60@

In this research, the above notation represents the rate of respiratory infections as an incidence rate for the population at risk. Since we know that overdispersion is present in the data, following Littel RC, et.al. [22] and SAS [23], we can rearrange the above notation to include a term for spatial variability *γ*_*i *_neighborhood random effects where the errors *ε *are correlated. *β*_0 _and *β*_1 _are the fixed effects and *λ*_*i *_the relative risk specific to each neighborhood and a random variable. Conditional on *λ*_*i *_the observed counts are independent Poisson variables with mean *E*_*i*_*λ*_*i*_, x_*i *_is the covariate measuring the poverty scores.

*λ*_*i *_= exp{*β*_0 _+ *β*_1_*x*_*i *_+ *γ*_*i*_} where *i *= 1, ... 439

The mixed model above is particularly appropriate when the assumptions of independence are violated, which is the case when spatial dependency is present. We then rearrange the terms above including the log link function log{*E*_*i*_}

log{*μ*_*i*_} = log{*E*_*i*_} + *β*_*0 *_+ *β*_1_*x*_1 _+ *γ*_*i*_

In the model above, and following Clayton and Kaldor (1987), who report the number of lip cancer cases registered during 1975–1980 in each of the 56 districts of Scotland [14], we denote *Y*_*i*_, *i *= 1,...*N*, *N *= 439 which are the neighborhoods of *Araucanía *region. We also report the estimates for the expected number of cases *E*_*i*_, accounting for the different age, gender, and ethnic distributions per neighborhood.

ri=YiEi
 MathType@MTEF@5@5@+=feaafiart1ev1aaatCvAUfKttLearuWrP9MDH5MBPbIqV92AaeXatLxBI9gBaebbnrfifHhDYfgasaacH8akY=wiFfYdH8Gipec8Eeeu0xXdbba9frFj0=OqFfea0dXdd9vqai=hGuQ8kuc9pgc9s8qqaq=dirpe0xb9q8qiLsFr0=vr0=vr0dc8meaabaqaciaacaGaaeqabaqabeGadaaakeaacqWGYbGCdaWgaaWcbaGaemyAaKgabeaakiabg2da9maalaaabaGaemywaK1aaSbaaSqaaiabdMgaPbqabaaakeaacqWGfbqrdaWgaaWcbaGaemyAaKgabeaaaaaaaa@3626@

Where *Y*_*i *_represents the observed event and *E*_*i *_is the expected count events per neighborhoods. The covariate, x_*i *_poverty scores spatially variates as displayed in Figure 3, suggesting thus that distance based covariate functions of poverty may be related with additional risk factors triggering respiratory infections; the variable *x*_*i *_are poverty scores measured by CAS-2/Family records.

The null hypothesis to be tested is that *β*_1 _= 0, indicating that poverty rates have no effects on respiratory infections once neighborhoods' spatial variations have been adjusted.

## Results

No published research articles have ever been linked poverty and disease in Chile's most deprived region. After a careful review of several international ISI databases, no articles were found on the subject. The present effort has taken advantage of information newly generated by the Chilean government and its release within a previously signed agreement of cooperation. Census data 1992 and 2002 now include ways to identify ancestral populations. Also, municipal poverty records *Ficha CAS-2/Familia *have been officially geo-referenced, and hospital discharge records allow – for the first time ever – the possibility to link a base map with patient information and individual records. This connection is necessary to test the relationship between poverty and disease at the individual and neighborhood level. While individual records and census data permit the generation of incidence rates of respiratory infections, geo-referenced base-maps (neighborhoods aggregations) further allow the incorporation of spatial methods and comparison with non-spatial Poisson models.

The results are presented in Table 4. A comparison of models (1) and (2) is presented to test the impact of overdispersion, as it can have significant effects on standard errors and therefore appropriate inference.

**Table 4:** Results of seven GLMMs Fit to the Respiratory Infections SMRs*

Models	β^0 MathType@MTEF@5@5@+=feaafiart1ev1aaatCvAUfKttLearuWrP9MDH5MBPbIqV92AaeXatLxBI9gBaebbnrfifHhDYfgasaacH8akY=wiFfYdH8Gipec8Eeeu0xXdbba9frFj0=OqFfea0dXdd9vqai=hGuQ8kuc9pgc9s8qqaq=dirpe0xb9q8qiLsFr0=vr0=vr0dc8meaabaqaciaacaGaaeqabaqabeGadaaakeaaiiGacuWFYoGygaqcamaaBaaaleaacqaIWaamaeqaaaaa@2F7E@	β^1 MathType@MTEF@5@5@+=feaafiart1ev1aaatCvAUfKttLearuWrP9MDH5MBPbIqV92AaeXatLxBI9gBaebbnrfifHhDYfgasaacH8akY=wiFfYdH8Gipec8Eeeu0xXdbba9frFj0=OqFfea0dXdd9vqai=hGuQ8kuc9pgc9s8qqaq=dirpe0xb9q8qiLsFr0=vr0=vr0dc8meaabaqaciaacaGaaeqabaqabeGadaaakeaaiiGacuWFYoGygaqcamaaBaaaleaacqaIXaqmaeqaaaaa@2F80@	σ^s2 MathType@MTEF@5@5@+=feaafiart1ev1aaatCvAUfKttLearuWrP9MDH5MBPbIqV92AaeXatLxBI9gBaebbnrfifHhDYfgasaacH8akY=wiFfYdH8Gipec8Eeeu0xXdbba9frFj0=OqFfea0dXdd9vqai=hGuQ8kuc9pgc9s8qqaq=dirpe0xb9q8qiLsFr0=vr0=vr0dc8meaabaqaciaacaGaaeqabaqabeGadaaakeaaiiGacuWFdpWCgaqcamaaDaaaleaacqWGZbWCaeaacqaIYaGmaaaaaa@3114@	σ^2 MathType@MTEF@5@5@+=feaafiart1ev1aaatCvAUfKttLearuWrP9MDH5MBPbIqV92AaeXatLxBI9gBaebbnrfifHhDYfgasaacH8akY=wiFfYdH8Gipec8Eeeu0xXdbba9frFj0=OqFfea0dXdd9vqai=hGuQ8kuc9pgc9s8qqaq=dirpe0xb9q8qiLsFr0=vr0=vr0dc8meaabaqaciaacaGaaeqabaqabeGadaaakeaaiiGacuWFdpWCgaqcamaaCaaaleqabaGaeGOmaidaaaaa@2FA5@	a^ MathType@MTEF@5@5@+=feaafiart1ev1aaatCvAUfKttLearuWrP9MDH5MBPbIqV92AaeXatLxBI9gBaebbnrfifHhDYfgasaacH8akY=wiFfYdH8Gipec8Eeeu0xXdbba9frFj0=OqFfea0dXdd9vqai=hGuQ8kuc9pgc9s8qqaq=dirpe0xb9q8qiLsFr0=vr0=vr0dc8meaabaqaciaacaGaaeqabaqabeGadaaakeaacuWGHbqygaqcaaaa@2E07@	*p value*
(1) PR	-2.9977 ± 0.1873	0.0348 ± 0.0370	-	-	-	<.0001
(2) PR+OD	-2.9977 ± 1.0671	0.0348 ± 0.0211	-	32.4638	-	0.0989
(3) Glimm (M)	-2.9977 ± 1.0671	0.0348 ± 0.0211	-	32.4638	-	0.0996
(4) Glimm (P)	-4.2609 ± 1.0310	0.0541 ± 0.0246	-	1.7241	-	0.0081
(5) GLMMI	-2.9977 ± 1.0671	0.0348 ± 6.4126	41.1208	32.4638	-	0.9957
(6) GLMME	-4.8934 ± 1.0778	0.0664 ± 0.0216	1.7673	-	0.5104	0.0023
(7) GLMMS	-4.8972 ± 1.0423	0.0666 ± 0.0208	1.7797	-	1.1166	0.0015

* The units of the spatial autocorrelation parameter a^
 MathType@MTEF@5@5@+=feaafiart1ev1aaatCvAUfKttLearuWrP9MDH5MBPbIqV92AaeXatLxBI9gBaebbnrfifHhDYfgasaacH8akY=wiFfYdH8Gipec8Eeeu0xXdbba9frFj0=OqFfea0dXdd9vqai=hGuQ8kuc9pgc9s8qqaq=dirpe0xb9q8qiLsFr0=vr0=vr0dc8meaabaqaciaacaGaaeqabaqabeGadaaakeaacuWGHbqygaqcaaaa@2E07@ are in kilometers. The p-value is based on testing *β*_1 _= 0

Model (5) Hessian Matrix negative

Estimates β^0
 MathType@MTEF@5@5@+=feaafiart1ev1aaatCvAUfKttLearuWrP9MDH5MBPbIqV92AaeXatLxBI9gBaebbnrfifHhDYfgasaacH8akY=wiFfYdH8Gipec8Eeeu0xXdbba9frFj0=OqFfea0dXdd9vqai=hGuQ8kuc9pgc9s8qqaq=dirpe0xb9q8qiLsFr0=vr0=vr0dc8meaabaqaciaacaGaaeqabaqabeGadaaakeaaiiGacuWFYoGygaqcamaaBaaaleaacqaIWaamaeqaaaaa@2F7E@ and β^1
 MathType@MTEF@5@5@+=feaafiart1ev1aaatCvAUfKttLearuWrP9MDH5MBPbIqV92AaeXatLxBI9gBaebbnrfifHhDYfgasaacH8akY=wiFfYdH8Gipec8Eeeu0xXdbba9frFj0=OqFfea0dXdd9vqai=hGuQ8kuc9pgc9s8qqaq=dirpe0xb9q8qiLsFr0=vr0=vr0dc8meaabaqaciaacaGaaeqabaqabeGadaaakeaaiiGacuWFYoGygaqcamaaBaaaleaacqaIXaqmaeqaaaaa@2F80@ are the same, -2.9977 and 0.0348, respectively, but for β^0
 MathType@MTEF@5@5@+=feaafiart1ev1aaatCvAUfKttLearuWrP9MDH5MBPbIqV92AaeXatLxBI9gBaebbnrfifHhDYfgasaacH8akY=wiFfYdH8Gipec8Eeeu0xXdbba9frFj0=OqFfea0dXdd9vqai=hGuQ8kuc9pgc9s8qqaq=dirpe0xb9q8qiLsFr0=vr0=vr0dc8meaabaqaciaacaGaaeqabaqabeGadaaakeaaiiGacuWFYoGygaqcamaaBaaaleaacqaIWaamaeqaaaaa@2F7E@ the associated standard error increases almost 5 times after adjusting for overdispersion. The *p-value *for model (2) is .0989 and becomes non significant at the .05 level. It is not possible, therefore, to reject the hypothesis that poverty is unrelated to respiratory infections. Model (3) also adjusts for overdispersion. We have used here the GLIMMIX Macro [30], essentially the results are the same as those obtained with SAS's PROC GENMOD procedure in model (2). Model (4) was generated with PROC GLIMMIX, one of SAS latest procedures. Whereas PROC GLIMMIX uses Residual Penalized Likelihood technique, the GLIMMIX Macro in model (3) uses REML as the estimation method. The 95% confidence interval of model (4) contains the estimate obtained in model (3), but with a much lower variance of σ^2
 MathType@MTEF@5@5@+=feaafiart1ev1aaatCvAUfKttLearuWrP9MDH5MBPbIqV92AaeXatLxBI9gBaebbnrfifHhDYfgasaacH8akY=wiFfYdH8Gipec8Eeeu0xXdbba9frFj0=OqFfea0dXdd9vqai=hGuQ8kuc9pgc9s8qqaq=dirpe0xb9q8qiLsFr0=vr0=vr0dc8meaabaqaciaacaGaaeqabaqabeGadaaakeaaiiGacuWFdpWCgaqcamaaCaaaleqabaGaeGOmaidaaaaa@2FA5@ = 1.7241, compared with 32.4638 of model (3), with *p-value *.0081 which is statistically significant. One may hypothesize whether the different estimation methods, residual PL and REML, have had effects in reducing estimated variances between model (4) and model (3). Model (5) is only included for reference. Although we obtained convergence, its Hessian matrix is negative. Models (6) and (7) include spatially correlated random effects, having no adjustment for overdispersion and using an exponential and a spherical correlation function correspondingly. Observing the estimates for the variance σ^s2
 MathType@MTEF@5@5@+=feaafiart1ev1aaatCvAUfKttLearuWrP9MDH5MBPbIqV92AaeXatLxBI9gBaebbnrfifHhDYfgasaacH8akY=wiFfYdH8Gipec8Eeeu0xXdbba9frFj0=OqFfea0dXdd9vqai=hGuQ8kuc9pgc9s8qqaq=dirpe0xb9q8qiLsFr0=vr0=vr0dc8meaabaqaciaacaGaaeqabaqabeGadaaakeaaiiGacuWFdpWCgaqcamaaDaaaleaacqWGZbWCaeaacqaIYaGmaaaaaa@3114@ 1.7673 and 1.7797, both models have very similar values, with *p-values *highly significant, .0023 and .0015 respectively. In model (6) β^0
 MathType@MTEF@5@5@+=feaafiart1ev1aaatCvAUfKttLearuWrP9MDH5MBPbIqV92AaeXatLxBI9gBaebbnrfifHhDYfgasaacH8akY=wiFfYdH8Gipec8Eeeu0xXdbba9frFj0=OqFfea0dXdd9vqai=hGuQ8kuc9pgc9s8qqaq=dirpe0xb9q8qiLsFr0=vr0=vr0dc8meaabaqaciaacaGaaeqabaqabeGadaaakeaaiiGacuWFYoGygaqcamaaBaaaleaacqaIWaamaeqaaaaa@2F7E@ = -4.8934 there is a standard error of 1.0778. The estimate of the slope β^1
 MathType@MTEF@5@5@+=feaafiart1ev1aaatCvAUfKttLearuWrP9MDH5MBPbIqV92AaeXatLxBI9gBaebbnrfifHhDYfgasaacH8akY=wiFfYdH8Gipec8Eeeu0xXdbba9frFj0=OqFfea0dXdd9vqai=hGuQ8kuc9pgc9s8qqaq=dirpe0xb9q8qiLsFr0=vr0=vr0dc8meaabaqaciaacaGaaeqabaqabeGadaaakeaaiiGacuWFYoGygaqcamaaBaaaleaacqaIXaqmaeqaaaaa@2F80@ is .0664, with a standard error of .0216.

The "test of fixed effects table" for model (6) (not displayed) shows the *F*-statistic for the test of H_0_: β^1
 MathType@MTEF@5@5@+=feaafiart1ev1aaatCvAUfKttLearuWrP9MDH5MBPbIqV92AaeXatLxBI9gBaebbnrfifHhDYfgasaacH8akY=wiFfYdH8Gipec8Eeeu0xXdbba9frFj0=OqFfea0dXdd9vqai=hGuQ8kuc9pgc9s8qqaq=dirpe0xb9q8qiLsFr0=vr0=vr0dc8meaabaqaciaacaGaaeqabaqabeGadaaakeaaiiGacuWFYoGygaqcamaaBaaaleaacqaIXaqmaeqaaaaa@2F80@ = 0 as *F *= 9.42 df = 448 with a *p*-value of .0023. Finally, we can conduct a Likelihood Ratio test of this model to that of the independent errors model. This model tests H_0_: *ρ *= 0 and σ12
 MathType@MTEF@5@5@+=feaafiart1ev1aaatCvAUfKttLearuWrP9MDH5MBPbIqV92AaeXatLxBI9gBaebbnrfifHhDYfgasaacH8akY=wiFfYdH8Gipec8Eeeu0xXdbba9frFj0=OqFfea0dXdd9vqai=hGuQ8kuc9pgc9s8qqaq=dirpe0xb9q8qiLsFr0=vr0=vr0dc8meaabaqaciaacaGaaeqabaqabeGadaaakeaaiiGacqWFdpWCdaqhaaWcbaGaeGymaedabaGaeGOmaidaaaaa@3085@ = 0 which is intended as a test of the existence of spatial variability. This test is a *χ*^2 ^test with 2 degrees of freedom, corresponding to the 2 parameters being tested. The resulting likelihood ratio *χ*^2 ^value is 1.655 – 1.094.1 = 550.9 and *p-value *= .05 Therefore, we conclude that significant spatial variability exists in these neighborhoods.

## Discussion

This article is an effort to provide a global approach to the links between poverty and disease, while comparing ethnic differences which are now identifiable using census data, individual medical records, and poverty information. The intensity of poverty has been captured by several dimensions such as income, education attained, employment, and housing conditions measured at an individual level by CAS-2/family scores. Previous information was later geo-referenced with GIS-based maps. The latter allowed us to collapse previous datasets into a seamless system of coordinates (x y) and neighborhoods to produce comparisons of conventional statistical models versus spatial statistical models. At the level of incidences (1) *Mapuches *had higher morbidity than non-*Mapuches*, particularly among children. The reverse happens among senior age-groups, where non-*Mapuches *had higher morbidities than *Mapuches *(2). For incidence rates in mortality, all diseases considered produced higher rates among *Mapuches *in all age-groups. Given the severity of these rates, a crucial element to be considered in future discussion is the survival of *Mapuches*. Additional data concerning their fertility rates and migratory processes among their women should highlight the discussion as to whether *Mapuches *are simply disappearing as an ethnic group. (3) Because of the clustered nature of respiratory infections and poverty found in the maps, we decided to test spatial dependence and treat the outcome variable within the framework of Generalized Linear Mixed Models (GLMM). Discussions involved not only issues of overdispersion, but also assumptions and different methods of covariate estimation using GLIMMIX Macro versus SAS's Proc GLIMMIX.

The limitations of the study involve those that are inherent to cross-sectional designs and/or that lack comparisons over time. Future findings should be able to include historical data in order to rule out seasonal/cyclical patterns or extreme – but circumstantial – observations. This is particularly important when small area events and incidence rates are modeled. Another limitation of the study is the exclusion of other important individual determinants of respiratory infections, such as the use of biomass fuels inside *Mapuche *huts, particularly in winter time. Other limitations in the study relate to the potential confounding of ethnicity [22]. In the former case, given that *Mapuches *have the worst poverty condition, it would not be poverty but the ethnic expression of poverty that would explain the original relationship between being poor and having respiratory infections. Ethnicity is an individual attribute, whereas poverty has been considered here as a contextual attribute of neighborhoods.

## Conclusion

Because of its unique conditions, the *Araucanía *region is suitable for testing an ongoing discussion which has captured much attention: the relationship between poverty/inequality and disease and the potential confounder of race as mediating between the two. The methods used to test this relationship – standardized morbidity/mortality rates comparing *Mapuche *versus non-*Mapuche *populations – have confirmed significant differentials in health for *Mapuche *children compared to non-*Mapuche *children and the reverse differential in senior age-groups. Diseases that end in deaths were much higher for *Mapuches *than non-*Mapuches*; this is true for all age-groups and all diseases. ARIs were no exception, and this research included additional efforts to model morbidity rates as the outcome variable with poverty rates at neighborhood level as the independent variable, while adjusting for spatial dependence. Several General Linear Mixed Models were included to test the original relationship. While the spatial regression model and the spherical and exponential covariance models confirmed the positive relationship between poverty and respiratory infections, they also tested the spatial variability among neighborhoods. Despite these promising findings, future research should incorporate multilevel methods and longitudinal designs as a way to establish additional controls to eliminate other potential confounding factors.

## Competing interests

The author(s) declare that they have no competing interests.
